# Interaction between gas cooking and *GSTM1* null genotype in bronchial responsiveness: results from the European Community Respiratory Health Survey

**DOI:** 10.1136/thoraxjnl-2013-204574

**Published:** 2014-03-10

**Authors:** André F S Amaral, Adaikalavan Ramasamy, Francesc Castro-Giner, Cosetta Minelli, Simone Accordini, Inga-Cecilie Sørheim, Isabelle Pin, Manolis Kogevinas, Rain Jõgi, David J Balding, Dan Norbäck, Giuseppe Verlato, Mario Olivieri, Nicole Probst-Hensch, Christer Janson, Jan-Paul Zock, Joachim Heinrich, Deborah L Jarvis

**Affiliations:** 1 Respiratory Epidemiology, Occupational Medicine and Public Health, National Heart and Lung Institute, Imperial College, London, UK; 2 MRC-PHE Centre for Environment & Health, London, UK; 3 Centre for Research in Environmental Epidemiology (CREAL), Barcelona, Spain; 4 Molecular and Population Genetics Laboratory, Wellcome Trust Centre for Human Genetics, University of Oxford, Oxford, UK; 5 Unit of Epidemiology and Medical Statistics, Department of Public Health and Community Medicine, University of Verona, Verona, Italy; 6 University of Bergen, Bergen, Norway; 7 Pédiatrie, CHU de Grenoble, Institut Albert Bonniot, INSERM, Grenoble, France; 8 Université Joseph Fourier, Grenoble, France; 9 Tartu University Hospital, Lung Clinic, Tartu, Estonia; 10 UCL Genetics Institute, University College London, London, UK; 11 Department of Medical Science, Occupational and Environmental Medicine, Uppsala University, Uppsala, Sweden; 12 Unit of Epidemiology & Medical Statistics, Dept. of Public Health & Community Medicine, University of Verona, Verona, Italy; 13 Unit of Occupational Medicine, University Hospital of Verona, Verona, Italy; 14 Swiss Tropical and Public Health Institute, Basel, Switzerland; 15 University of Basel, Basel, Switzerland; 16 Department of Medical Sciences, Respiratory Medicine and Allergology, Uppsala University, Uppsala, Sweden; 17 Universitat Pompeu Fabra (UPF), Barcelona, Spain; 18 CIBER Epidemiologia y Salud Pública (CIBERESP), Barcelona, Spain; 19 Helmholtz Zentrum München, German Research Centre for Environmental Health, Institute of Epidemiology I, Neuherberg, Germany

**Keywords:** Asthma

## Abstract

**Background:**

Increased bronchial responsiveness is characteristic of asthma. Gas cooking, which is a major indoor source of the highly oxidant nitrogen dioxide, has been associated with respiratory symptoms and reduced lung function. However, little is known about the effect of gas cooking on bronchial responsiveness and on how this relationship may be modified by variants in the genes *GSTM1*, *GSTT1* and *GSTP1*, which influence antioxidant defences.

**Methods:**

The study was performed in subjects with forced expiratory volume in one second at least 70% of predicted who took part in the multicentre European Community Respiratory Health Survey, had bronchial responsiveness assessed by methacholine challenge and had been genotyped for *GSTM1*, *GSTT1* and *GSTP1*-rs1695. Information on the use of gas for cooking was obtained from interviewer-led questionnaires. Effect modification by genotype on the association between the use of gas for cooking and bronchial responsiveness was assessed within each participating country, and estimates combined using meta-analysis.

**Results:**

Overall, gas cooking, as compared with cooking with electricity, was not associated with bronchial responsiveness (β=−0.08, 95% CI −0.40 to 0.25, p=0.648). However, *GSTM1* significantly modified this effect (β for interaction=−0.75, 95% CI −1.16 to −0.33, p=4×10^−4^), with *GSTM1* null subjects showing more responsiveness if they cooked with gas. No effect modification by *GSTT1* or *GSTP1*-rs1695 genotypes was observed.

**Conclusions:**

Increased bronchial responsiveness was associated with gas cooking among subjects with the *GSTM1* null genotype. This may reflect the oxidant effects on the bronchi of exposure to nitrogen dioxide.

**Key messages**

**What is the key question?**

- Is the relationship of bronchial responsiveness with use of gas for cooking modified by variants in oxidative stress-related genes (*GSTM1*, *GSTT1*, *GSTP1*)?

**What is the bottom line?**

- In subjects with *GSTM1* null genotype, increased bronchial responsiveness was associated with gas cooking, which may reflect the oxidant effects on the bronchi of exposure to gas-derived pollutants.

**Why read on?**

- Since increased bronchial responsiveness is a characteristic feature of asthma, the present findings may help in understanding why some individuals may present asthma-related symptoms when cooking with gas while others do not.

## Introduction

Gas cooking is a major source of indoor nitrogen dioxide and, to a lesser extent, of fine particles.^1^ ^2^ Use of gas for cooking has been inconsistently associated with respiratory symptoms, including wheeze and exacerbation of asthma, and reduced lung function suggestive of airways obstruction,^3–6^ and only a few studies have examined associations with bronchial responsiveness (BR). In a study of 324 Montreal school children, those exposed to gas cooking were more likely to have increased BR.^7^ In the Dutch arm of the European Community Respiratory Health Survey (ECRHS), which involved 1921 adults, gas cooking was also associated with increased BR, but only among those with high total immunoglobulin E (IgE) levels.^8^ In contrast, in a study of 929 eight-year-old Dutch children, exposure to gas cooking was not associated with BR.^9^

Nitrogen dioxide is an oxidant species that induces upregulation of the expression of T helper type 2 cell cytokines and ICAM1 as well as neutrophilic inflammation in bronchial epithelium.^10^ ^11^ Changes in air particle number concentrations have also been linked to airway inflammation.^12^ The extent to which these and other pollutants cause lung damage and inflammation is dependent on the efficacy of internal antioxidant defences and detoxification mechanisms. Glutathione-S-transferases are a group of phase II enzymes involved in the detoxification of xenobiotics in the lung.^13^ These enzymes play a role in the protection against oxidative stress since they influence the levels of glutathione, an important non-enzymatic antioxidant in the lung.^14^ ^15^ Variants in genes encoding glutathione-S-transferase mu 1 (GSTM1), theta 1 (GSTT1) and pi 1 (GSTP1) have been linked to decreased lung function and progression from increased BR to asthma,^16–18^ suggesting that these variants contribute to increased susceptibility to oxidative stress. Thus, the aim of study was to assess whether genetic variants in *GSTM1* (null genotype), *GSTT1* (null genotype) and *GSTP1* (rs1695[G]) modify the association between gas cooking and BR.

## Methods

### Participants

Data included in the following analysis were collected from subjects participating in the ECRHS, an international multicentre cohort study designed to identify risk factors for asthma.^19^ In the first survey (ECRHS I), subjects were randomly recruited from community-based sampling frames in each centre after completing a short postal screening questionnaire. A random sample of responders to the postal survey completed an interviewer-led questionnaire between 1992 and 1994 (‘random’ group). A smaller sample of participants with symptoms highly suggestive of asthma, but who had not been randomly selected to take part, was also invited for clinical assessment (‘enriched’ group). Approximately 8 years later, subjects who had participated in the clinical investigations during the first survey were invited for further questionnaires and blood sampling for genotyping (ECRHS II: 1999–2002). The main analysis herein presented is based on data collected in ECRHS II.

Ethical approval for the study from local research ethics committees and written consent from subjects were obtained.

### Genotyping

**Figure 1:**
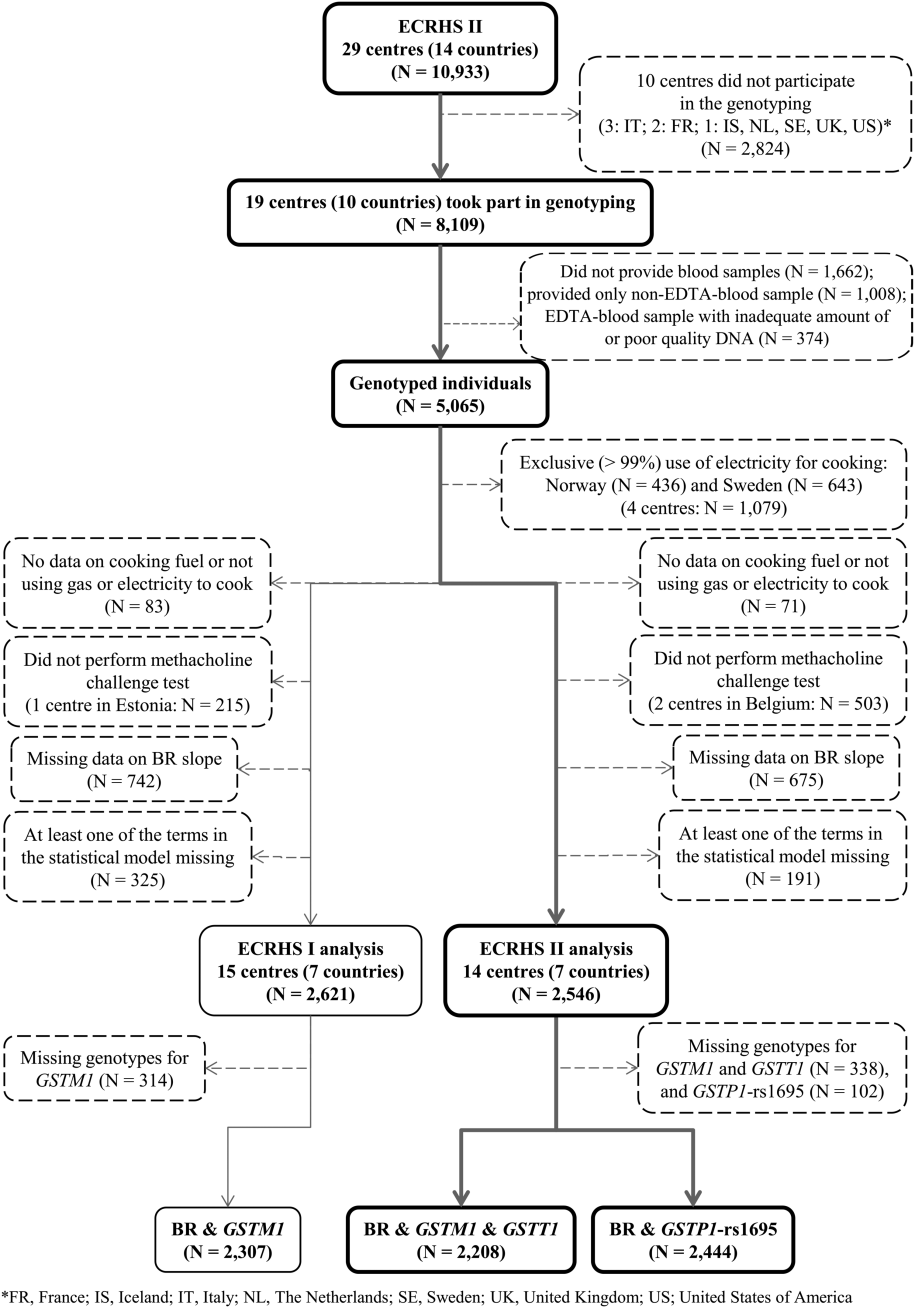
Flow diagram showing the selection of subjects included in the analysis.

In total, 19 of 29 centres (8109 out of 10 933 subjects) in ECRHS II collected blood samples for genotyping (figure 1). However, not all subjects provided blood samples, and some of the collected samples had inadequate amount of or poor quality DNA. Only 5065 out of the 8109 subjects were genotyped for *GSTM1*, *GSTP1* and *GSTT1*. Genotyped subjects were slightly older than those who were not genotyped, and as there were some between-country differences in response to genotyping, the distribution by country was not the same between the two groups (see online supplementary T1). DNA was extracted from blood samples using a commercial kit (Puregene, Gentra Inc., MN, USA). At the Centre for Genomic Regulation (Barcelona, Spain), *GSTM1* and *GSTT1* null genotypes were determined by PCRs, and *GSTP1* polymorphism (rs1695—Ile105Val) was genotyped using a specific pyrosequencing assay.^20^ Genotype frequencies at *GSTP1*-rs1695 deviated from Hardy–Weinberg equilibrium (HWE) in France and Germany. HWE could not be calculated for *GSTM1* and *GSTT1* because our data did not distinguish between one and two copies of the variant allele. Population stratification was assessed with 26 unlinked markers using the genomic control approach and the EIGENSTRAT software. There was no evidence of relevant population stratification.^20^

### Exposure to gas cooking

Participants were asked, “What kind of stove do you mostly use for cooking?” Subjects who answered ‘gas (gas from the mains)’ or ‘gas (gas from bottles or other non-mains source)’ were classified as being exposed to gas cooking. Those who answered ‘electric’ or ‘microwave’ were classified as the reference group. Subjects in four centres in Norway (N=436) and Sweden (N=643) were not included in the analysis because the use of gas for cooking in these countries is extremely low (<1%). Subjects who used other types of stove, such as ‘coal, coke or wood (solid fuel)’ or ‘paraffin (kerosene)’, were also excluded (<2% of participants in the analysis, N=71).

### Bronchial responsiveness

BR was assessed by methacholine bronchial challenge test as previously described.^21^ To assess the baseline forced expiratory volume in one second (FEV1) and the forced vital capacity (FVC), each participant was allowed nine attempts to provide at least two technically acceptable expiratory manoeuvres. Subjects with FEV1 at least 70% of predicted, and more than 1.5 L, underwent bronchial challenge unless they had specific contraindications. Bronchial challenge was conducted with increasing amounts of methacholine up to a cumulative dose of 1 mg, with the methacholine solution being administered via a dosimeter. BR was defined by the slope of the dose–response curve obtained with the methacholine test. The ‘slope’ was estimated as rate of change of FEV1 against methacholine dose and, in order to satisfy the assumption of normality and homogeneity of variance, transformed to 100/(log-slope+10).^21^ ^22^ A low slope is indicative of high BR. In addition, subjects who experienced more than a 20% fall in FEV1 after inhalation of 1 mg of methacholine were identified, that is, those with PD20 <1 mg. In ECRHS II, two centres in Belgium (N=503) did not perform the methacholine challenge test and their data were excluded.

### Statistical analysis

#### Main analysis: ECRHS II

To assess whether genetic variants in *GSTM1*, *GSTT1* and *GSTP1* modify the association between gas cooking and BR, linear regression models with the slope as the continuous dependent variable were fitted for each of the three genes and a gene–gas cooking interaction term was entered in the models. *GSTM1* and *GSTT1* were modelled as dichotomous variables (‘null’ vs ‘present’), whereas *GSTP1*-rs1695[G] was modelled *per* number of minor alleles (0, 1, 2) under an additive mode of inheritance. The coefficient (β) for the interaction term between each of the genes and gas cooking was estimated for the whole sample (‘random’ and ‘enriched’) in each country and then combined using a random effects meta-analysis.^23^ This was repeated adding in the model a term for the type of sample. The analysis was also repeated for both sexes, separately.

Potential confounders considered a priori to be relevant included the following: age, sex, height, smoking in pack-years, specific IgE titre (cat, house dust mite, Timothy-grass, *Cladosporium herbarum*), total IgE, baseline FEV1 expressed as a standardised difference from an internally derived sex-specific predicted value and baseline FEV1 as a percentage of FVC. An age–sex interaction^24^ and a gene–smoking interaction, as suggested in the literature,^21^ were also considered. In addition, the interaction between each genetic variant and BR was further adjusted for the other genetic variants. After excluding subjects with missing slope, genotype and any of the potential confounders included in the models, the total sample consisted of 2208 for models assessing the interaction between gas cooking and *GSTM1* or *GSTT1*, and 2444 for models assessing the interaction with *GSTP1* (figure 1). Statistical tests were two-sided, and results were considered significant when nominal p≤0.05. Statistical analyses were performed using STATA/IC V.12.1.^25^

### Sensitivity and post hoc analyses

Details on the sensitivity and post hoc analyses are provided in the online supplementary material.

## Results

### Main results: ECRHS II

In the present study, both sexes were equally represented, most subjects were ever smokers (42.9% were lifetime non-smokers), most were from Spain, France and Germany, and cooking with gas was more prevalent than cooking with electricity (table 1). *GSTM1* and *GSTT1* were not present in just above 50% and approximately 20% of the subjects, respectively. Close to half of the subjects were heterozygous for *GSTP1*-rs1695.

**Table 1:** Characteristics of subjects from the two surveys of the European Community Respiratory Health Survey (ECRHS) included in the present analysis

	ECRHS I (N=2621)*	ECRHS II (N=2546)*
Age, mean (SD)	34.2 (7.2)	42.2 (7.2)
Sex (%)		
Males	50.4	49.4
Females	49.6	50.6
Smoking status (%)		
Never smoker	42.9%	42.9%
Ever smoker	57.1%	57.1%
Smoking pack-years, mean (SD)	7.8 (12.5)	11.0 (17.6)
Country (%)		
Australia	10.7	11.5
Belgium	11.8	–
Estonia	–	5.1
France	15.5	17.1
Germany	13.1	13.0
Spain	30.4	35.5
Switzerland	8.8	9.4
UK	9.7	8.4
Cooking fuel (%)		
Electricity	33.5	49.4
Gas	66.5	50.6
*GSTM1* genotype (%)†		
Null	51.1	51.2
Present	48.9	48.8
*GSTT1* genotype (%)†		
Null	19.8	20.2
Present	80.2	79.8
*GSTP1*-rs1695 genotype (%)‡		
AA	42.8	41.7
AG	48.3	49.0
GG	8.9	9.3
Bronchial responsiveness, log-slope, mean (SD)	7.6 (2.3)	7.4 (2.3)
Fall of 20% in FEV1 after inhalation of 1 mg methacholine, PD20 (%)		
No	84.2	83.1
Yes	15.8	16.9
Baseline FEV1, mean (SD)	3.7 (0.8)	3.6 (0.8)
Baseline FVC, mean (SD)	4.6 (1.0)	4.4 (1.0)

*After exclusion of subjects with missing data on cooking fuel or not using gas or electric stove, who did not perform the methacholine challenge test or had missing data on BR slope, and those with missing data on at least one of the terms in the statistical model.

†314 subjects from ECRHS I and 338 subjects from ECRHS II have missing data on *GSTM1* and *GSTT1* genotypes.

‡151 subjects from ECRHS I and 102 subjects from ECRHS II have missing data on *GSTP1*-rs1695 genotype.

**Figure 2:**
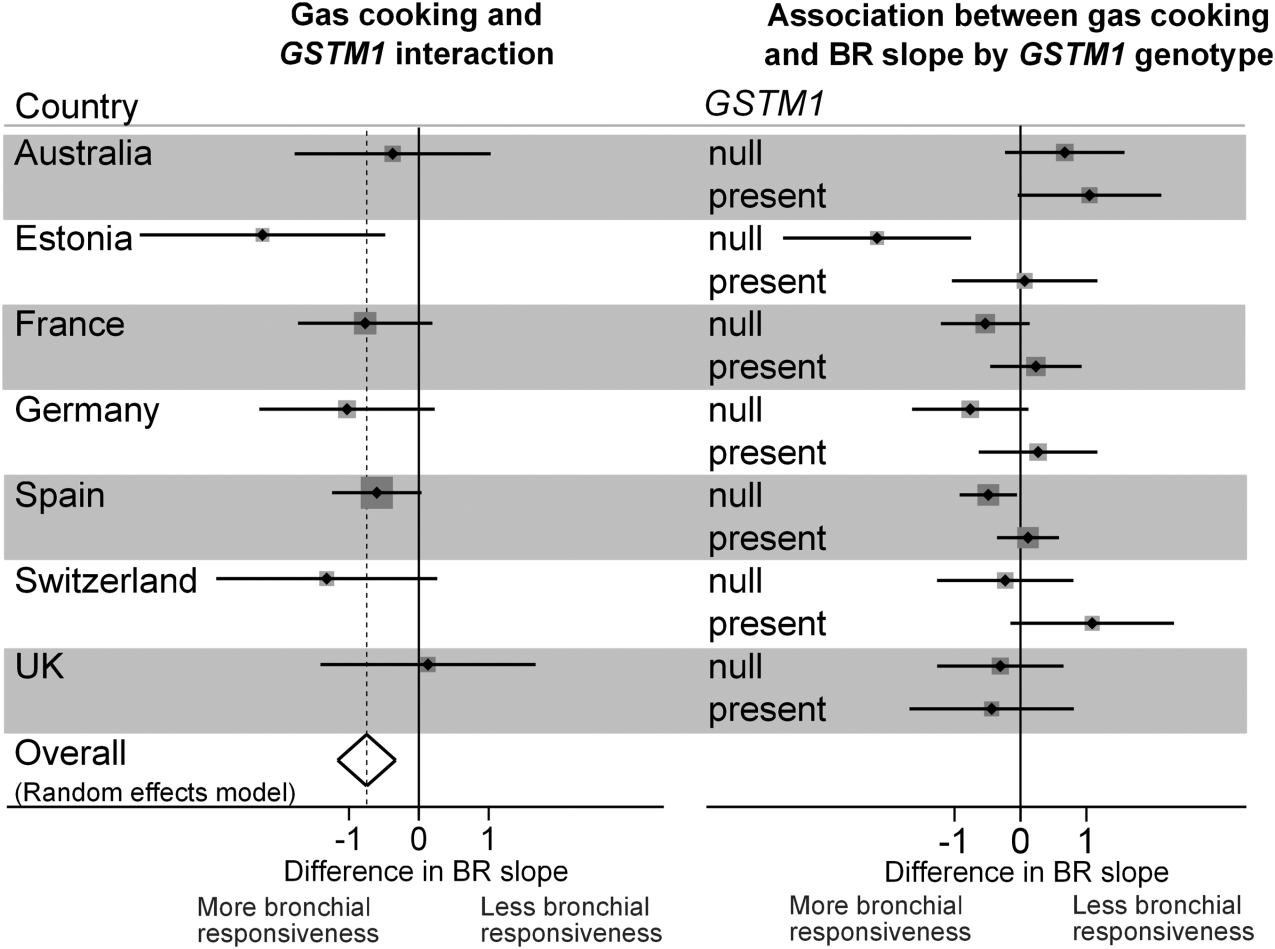
Interaction between gas cooking and *GSTM1* on bronchial responsiveness, and association between gas cooking and bronchial responsiveness according to *GSTM1* genotype, in the European Community Respiratory Health Survey II.

None of the three genetic variants was significantly associated with BR (see online supplementary T2). Overall, gas cooking, versus cooking with electricity, was also not significantly associated with BR (β=−0.08, 95% CI −0.40 to 0.25, p=0.648). However, this association was different depending on *GSTM1*, with *GSTM1* null subjects showing a strong significant association of increased BR with use of gas for cooking (β for interaction=−0.75, 95% CI −1.16 to −0.33, p=4×10^−4^, Bonferroni-corrected p=0.017) (table 2 and figure 2). There was no evidence of heterogeneity between countries (I^2^=0%, p=0.521). The interaction was present among males (β for interaction=−0.79, 95% CI −1.63 to 0.06, p=0.067) and females (β for interaction=−0.79, 95% CI −1.40 to −0.19, p=0.010). Adjustment of these models for the type of sample, a priori confounders and for the other genes did not materially alter the estimates for the interaction (see online supplementary T3). The association of BR with use of gas for cooking was not modified by *GSTT1* or *GSTP1*-rs1695 genotypes (table 2; see online supplementary T4).

**Table 2:** Estimates for the interaction between gas cooking and genetic variants in *GSTM1*, *GSTT1* and *GSTP1* on bronchial responsiveness in the European Community Respiratory Health Survey II

Genetic variant	Whole sample			‘Random’ sample			‘Enriched’ sample*		
	N	β (95% CI)	p Value	N	β (95% CI)	p Value	N	β (95% CI)	p Value
*GSTM1* null	2208	−0.75 (−1.16 to −0.33)	4×10^−4^	1843	−0.81 (−1.34 to −0.28)	0.003	365	−0.93 (−2.01 to 0.14)	0.088
*GSTT1* null	2208	−0.02 (−0.54 to 0.50)	0.929	1843	−0.27 (−0.83 to 0.28)	0.336	365	0.74 (−0.57 to 2.04)	0.270
*GSTP1*-rs1695	2444	0.03 (−0.41 to 0.46)	0.905	2018	0.01 (−0.45 to 0.48)	0.954	426	0.31 (−0.48 to 1.11)	0.441

*France and Switzerland were considered as one group due to small numbers in those two countries.

### Sensitivity analysis

The interaction between gas cooking and *GSTM1* was present and of the same magnitude in the ‘random’ sample and the smaller sample that was enriched in symptomatic subjects (table 2). The estimate for the interaction was similar in those who had gas hobs (without gas ovens), who used gas mains, who cooked every day in the last 4 weeks, even in a ventilated kitchen, and stronger in those who had gas hobs with gas ovens, who used bottled gas and who cooked every day in the last 4 weeks in an unventilated kitchen (table 3). When the analysis was restricted to lifetime non-smokers only (N=936), the magnitude of interaction reduced slightly, although the direction remained the same (β for interaction=−0.42, 95% CI −1.07 to 0.24, p=0.214).

**Table 3:** Estimates for the interaction between gas cooking and *GSTM1* null genotype on bronchial responsiveness in the European Community Respiratory Health Survey II, restricting the analysis according to cooking characteristics

Cooking characteristics	N	β (95% CI)	p Value
Gas hobs (without gas oven) vs electricity	1565	−0.53 (−1.06 to −0.001)	0.050
Gas hobs (with gas oven) vs electricity	1393	−0.85 (−1.43 to −0.27)	0.004
Bottled gas vs electricity	814	−1.59 (−2.98 to −0.20)	0.025
Gas mains vs electricity	1934	−0.60 (−1.04 to −0.15)	0.009
Cooked every day in the last four weeks	1578	−0.72 (−1.32 to −0.12)	0.018
Cooked every day in the last four weeks (ventilated kitchen)	1205	−0.50 (−1.04 to 0.04)	0.071
Cooked every day in the last four weeks (unventilated kitchen)	373	−1.47 (−2.54 to −0.40)	0.007

When BR was considered as PD20, the prevalence of increased BR in some centres was very low. Using this outcome, no statistically significant interaction was observed (OR for interaction=1.29, 95% CI 0.64 to 2.62, p=0.478).

### Post hoc analysis

Some analyses were repeated using all available data obtained at ECRHS I (ie, including participants from Belgium). The interaction between gas cooking and *GSTM1* was not present (β for interaction=−0.001, 95% CI −0.45 to 0.44, p=0.996), and there was some evidence of minor heterogeneity between countries in this relationship (I^2^=10.6%, p=0.349). As this post hoc analysis included participants from Belgium (who did not conduct BR measures at the follow-up), the analysis was repeated excluding participants from that country but still no interaction was observed (β for interaction=0.12, 95% CI −0.35 to 0.58, p=0.628). Exclusion of Spain (country contributing the most to heterogeneity of results at ECRHS I) did not alter results (β for interaction=−0.19, 95% CI −0.67 to 0.30, p=0.452). Of note, Spain showed the largest change in the use of gas cooking between the two surveys (84.2% to 54.8%, online supplementary T5). Some subjects changed cooking fuel between surveys (gas to electricity: N=483, of whom 35% were from Spain; electricity to gas: N=139). Restriction of the analysis to those who used the same fuel at baseline and follow-up still did not show an interaction between gas cooking and *GSTM1* in the ECRHS I. As in the second survey, interactions between gas cooking and *GSTT1* and *GSTP1*-rs1695 were not observed in the baseline survey (p>0.05; data not shown).

Overall, there was no evidence that use of gas over the approximately 8 years of follow-up was associated with greater increases in BR (difference in change in slope comparing gas with electricity: −0.002, 95% CI −0.51 to 0.51, p=0.995, N=1150). However, participants with the *GSTM1* null genotype and who cooked with gas, compared with those who cooked with electricity, throughout the period of follow-up showed some sign of greater increase in BR (β for the interaction between gas cooking and *GSTM1* on change in BR=−0.25, 95% CI −0.88 to 0.38, p=0.437, N=1150). There was no evidence that *GSTT1* or *GSTP1*-rs1695 genotypes modified the change in BR due to the use of gas (p>0.05; data not shown).

## Discussion

In this population-based study of adults of European ancestry and FEV1 at least 70% of predicted, the association of BR with gas cooking was different depending on *GSTM1*, that is, gas cooking was associated with increased BR among subjects with the null genotype for *GSTM1*, but not among carriers of that gene. Furthermore, the interaction was stronger among those who cooked in conditions where higher exposure levels are expected (ie, with gas oven and in unventilated kitchens). To our knowledge, this is the first study to assess and report this interaction.

One of the strengths of the present study is its large population sample derived from several areas of Europe. There was some loss to follow-up between the two surveys but little reason to believe that this would result in the detection of false gene–environment interactions as loss to follow-up in subjects with respiratory symptoms and cooking with gas, or electricity, are unlikely to be related to genetic characteristics. The general decrease in prevalence of cooking with gas across countries, from the first to the second survey, probably reflects a change in house building trends and is also unlikely due to genetic makeup. A strength of the study was the use of a standardised protocol across participating centres to perform methacholine bronchial challenge tests.^22^ The continuous non-censored slope obtained from the bronchial challenge tests performs better than PD20 in indicating BR.^26^

Despite the fact we were underpowered to detect interactions between gas cooking and *GSTT1* and *GSTP1*, we had enough power (>90%) to detect an interaction with *GSTM1* in both surveys. However, we only observed it in ECRHS II. This may be due to heterogeneity between countries being lower in ECRHS II or to lack of information on some environmental exposure that might have confounded the effect of gas cooking in the first survey. It may also be due to better insulated houses and consequently increased concentration of indoor gases in the second survey, but we do not have data to confirm or reject this argument. We could not detect within-survey differences between age groups, although there was some evidence of increasing BR due to gas cooking among older participants with *GSTM1* null genotype, when comparing the second with the first survey (data not shown). While the change in cooking fuel between the two surveys may, in part, explain that the interaction was statistically significant only in the most recent survey, we should not exclude the potential role of other genes, epigenetic mechanisms and cellular phenomena during normal ageing. Adjustment for *GSTT1* and *GSTP1*-rs1695 did not make a difference on the estimates for the interaction between *GSTM1* and gas cooking, nor did adjusting for *NQO1*-rs1800566, which has been proposed to interact with *GSTM1* and air pollutants on lung function (data not shown).^27^ Detrimental effects of gene dosage changes (eg, deletion, duplication) may eventually be avoided through compensation at the transcriptional, post-transcriptional and protein levels.^28^ ^29^ However, the efficiency of this phenomenon may decrease with ageing, and manifestation of the gene dosage changes may arise late in life. This is supported by evidence from studies on autosomal recessive diseases and mitochondrial disorders showing that even among subjects with inherited causal mutations a proportion may live several years or decades without manifesting the disease.^30^ ^31^ It is also possible that BR is affected by ageing-related decline in baseline lung function; however, we did not observe a relevant change in the estimate for the interaction after adjusting for FEV1 and FEV1/FVC ratio.

There is evidence of the influence of interactions between *GSTM1* and some environmental pollutants on asthma and airway obstruction. In a study of school children, prevalence of asthma was associated with maternal smoking during pregnancy, but only among children with *GSTM1* deletion.^24^ In a study of adolescents and young adults with asthma, it was reported that exposure to environmental tobacco smoke significantly reduces peak expiratory flow rate among subjects with no copy of *GSTM1*, but not among carriers of at least a copy of that gene.^32^ In a randomised controlled trial with asthmatic children and irrespective of the treatment being studied, forced expiratory flow was significantly reduced due to exposure to ozone among children with no copy of *GSTM1,* but not among those with at least a copy of *GSTM1*.^33^

*GSTM1* is located on chromosome 1 where it encodes a phase II enzyme involved in the detoxification of electrophilic xenobiotics, by conjugation with glutathione, and the protection against oxidative and nitrosative stress.^13–15^ Knockdown of *GSTM1* in normal human bronchial epithelial cells has shown that this gene may regulate diesel exhaust particle-induced expression of IL-8 and IL-1β by modulation of reactive oxygen species.^34^ In vitro and in vivo data have shown that knockout mice for *gstm1* have low ability to conjugate 1,2-dichloro-4-nitrobenzene with glutathione.^35^ Thus, it is biologically plausible that the lack of *GSTM1*, common in approximately half of the population of European ancestry, may lead to an increased susceptibility to the effects of gas cooking, which is the main source of indoor nitrogen dioxide and particulate matter.^1^ ^2^

In summary, increased BR was associated with gas for cooking among subjects with the null genotype for *GSTM1*. This may be an indication of the oxidant effects on the bronchi of exposure to nitrogen dioxide originating from cooking with gas. Further larger studies are recommended to confirm this finding and better understand its mechanism of action.

## Supplementary Material

Web supplement
