# Supplementary material for: Interaction between gas cooking and GSTM1 null genotype in bronchial responsiveness: results from the European Community Respiratory Health Survey
Source: Thorax. 2014 Mar 10;69(6):558–64. doi: 10.1136/thoraxjnl-2013-204574 (PMC4033138; doi:10.1136/thoraxjnl-2013-204574)
Supplement: Web supplement [file thoraxjnl-2013-204574-s1.pdf]

Online supplement

Title

Interaction between gas cooking and *GSTM1* null genotype in bronchial responsiveness: Results from the European Community Respiratory Health Survey

Corresponding author

André F S Amaral

Respiratory Epidemiology and Public Health

National Heart and Lung Institute, Imperial College London

Emmanuel Kaye Building, 1B Manresa Road

London SW3 6LR (UK)

Tel: +44 (0) 207 594 7940

Email: a.amaral@imperial.ac.uk

Authors and affiliations

André F S Amaral[1,2], Adaikalavan Ramasamy[1], Francesc Castro-Giner[3,4], Cosetta Minelli[1], Simone Accordini[5], Inga-Cecilie Sørheim[6], Isabelle Pin[7,8], Manolis Kogevinas[3], Rain Jõgi[9], David J Balding[10], Dan Norbäck[11], Giuseppe Verlato[12], Mario Olivieri[13], Nicole Probst-Hensch[14,15], Christer Janson[16,17], Jan-Paul Zock[3,18,19], Joachim Heinrich[20], Deborah L Jarvis[1,2]

[1]Respiratory Epidemiology and Public Health, National Heart and Lung Institute, Imperial College, London, UK

[2]MRC-PHE Centre for Environment & Health, London, UK

[3]Centre for Research in Environmental Epidemiology (CREAL), Barcelona, Spain

[4]Molecular and Population Genetics Laboratory, Wellcome Trust Centre for Human Genetics, University of Oxford, Oxford, UK

[5]Unit of Epidemiology and Medical Statistics, Department of Public Health and Community Medicine, University of Verona, Verona, Italy

[6]University of Bergen, Bergen, Norway

[7]Pédiatrie, CHU de Grenoble, Institut Albert Bonniot, INSERM, Grenoble

[8]Université Joseph Fourier, Grenoble, France

[9]Tartu University Hospital, Lung Clinic, Tartu, Estonia

[10]UCL Genetics Institute, University College London, London, UK

[11]Department of Medical Science, Occupational and Environmental Medicine, Uppsala University, Uppsala, Sweden

[12]Unit of Epidemiology & Medical Statistics, Dept. of Public Health & Community Medicine, University of Verona, Verona, Italy

[13]Unit of Occupational Medicine, University Hospital of Verona, Verona, Italy

[14]Swiss Tropical and Public Health Institute, Basel, Switzerland

[15]University of Basel, Basel, Switzerland

[16]Department of Medical Sciences, Respiratory Medicine and Allergology, Uppsala University, Uppsala, Sweden

[17]Department of Medical Science, Respiratory Medicine and Allergology, Uppsala University, Sweden

[18]Municipal Institute of Medical Research (IMIM-Hospital del Mar), Barcelona, Spain

[19]CIBER Epidemiologia y Salud Pública (CIBERESP), Barcelona, Spain

[20]Helmholtz Zentrum München, German Research Centre for Environmental Health, Institute of Epidemiology I, Neuherberg, Germany

Table of contents **Page**

Supplementary methods ……………..……………………………………………………..…….4

Supplementary T1. Characteristics of non-genotyped and genotyped subjects from the European Community Respiratory Health Survey ……………….……………………..…….5

Supplementary T2. Estimates for the association between genetic variants in *GSTM1*, *GSTT1*, and *GSTP1* and bronchial responsiveness in the European Community Respiratory Health Survey II ………………………………..……………………………………….6

Supplementary T3. Estimates for the interaction between gas cooking and *GSTM1* on bronchial responsiveness in the European Community Respiratory Health Survey II, after adjustment for potential confounders considered *a priori* to be of relevance ……………………….………..……………………………………………...…….7

Supplementary T4. Estimates for the interaction between gas cooking and genetic variants in *GSTM1*, *GSTT1*, and *GSTP1* on bronchial responsiveness, by sex, in the European Community Respiratory Health Survey II ………………………………..……………….8

Supplementary T5. Prevalence of gas cooking, as compared to cooking with electricity, and *GSTM1* null genotype in the European Community Respiratory Health Survey …...……….9

Supplementary T6. Characteristics of subjects from the European Community Respiratory Health Survey not included versus those included in the final analysis …..………..…….10

Supplementary T7. Characteristics of genotyped subjects from the European Community Respiratory Health Survey not included versus those included in the final analysis ………11

Supplementary methods

Sensitivity analysis

The interaction between gas cooking and *GSTM1* null genotype on BR was estimated for the ‘random’ and ‘enriched’ samples separately. Assuming that the association between gas cooking and BR may be dependent on the type of cooking appliance, type of gas, frequency of use, and kitchen ventilation while cooking, a set of analyses were carried out to exclude any potential influence by these variables. Ever smokers were excluded to minimize the potential effect of residual confounding by smoking. Because it has been suggested that *GSTM1* in combination with *NQO1*-rs1800566 may interact with air pollutants,[1] the analysis was repeated adjusting for a variable that pooled the *GSTM1* null genotype with the *NQO1*-rs1800566[CC]. An extra analysis was performed using PD20 as the outcome in a logistic regression model, adjusted for the same potential confounders as in the linear regression models, to examine the interaction of gas cooking with *GSTM1* on BR.

Post hoc analysis

To check for consistency between the baseline (1992-1994, ECRHS I) and follow-up (2000-2002, ECRHS II, when samples for genotyping were collected) of ECRHS, the interaction between gas cooking and each of the three genes was assessed using data collected at the baseline survey (ECRHS I). In a longitudinal analysis to examine change in BR over time, a linear generalized estimating equations model, with exchangeable correlations, was built with data from both surveys.

Reference

1 Minelli C, Wei I, Sagoo G, et al. Interactive effects of antioxidant genes and air pollution on respiratory function and airway disease: a HuGE review. Am J Epidemiol 2011;**173**:603-20.

Supplementary T1. Characteristics of non-genotyped and genotyped subjects from the European Community Respiratory Health Survey.

	**Non-genotyped subjects (N = 3,044)**	**Genotyped subjects (N = 5,065)**	***P*** *
**Age (mean, standard deviation)**	42.2 (7.1)	42.7 (7.2)	0.002
**Sex (%)**			
Males	45.9%	47.8%	0.087
Females	54.1%	52.2%	
**Smoking status (%)**[a]			
Never smoker	45.0%	44.1%	0.456
Ever smoker	55.0%	55.9%	
**Smoking pack-years (mean, standard deviation)**[a]	9.7 (15.3)	10.5 (17.4)	0.065
**Country (%)**[b]			
Australia	9.7%	6.7%	< 0.001
Belgium	7.0%	10.0%	
Estonia	3.3%	4.5%	
France	10.1%	10.8%	
Germany	5.0%	8.7%	
Norway	5.3%	8.6%	
Spain	24.2%	23.0%	
Sweden	19.0%	12.7%	
Switzerland	5.5%	7.9%	
UK	10.9%	7.1%	
**Cooking fuel (%)**			
Electricity	59.1%	60.6%	0.186
Gas	40.9%	39.4%	
**Bronchial responsiveness, log-slope (mean, standard deviation)**	7.4 (2.2)	7.5 (2.2)	0.279

P was calculated using Pearson's chi-square test for count data and Student's t-test for continuous data.

[a]One hundred and sixty four non-genotyped and two hundred and eighty four genotyped subjects have missing information on smoking status and pack-years.

[b]Norway and Sweden were excluded from the analysis for exclusive cooking with electricity.

Supplementary T2. Estimates for the association between genetic variants in *GSTM1*, *GSTT1*, and *GSTP1* and bronchial responsiveness in the European Community Respiratory Health Survey II.

**Genetic variant**	**N**	**Beta (95% CI)**	***P***
***GSTM1* null**	2,208	0.003 (-0.19 to 0.19)	0.978
***GSTT1* null**	2,208	-0.07 (-0.36 to 0.22)	0.644
***GSTP1*-rs1695**	2,444	0.06 (-0.08 to 0.20)	0.424

Supplementary T3. Estimates for the interaction between gas cooking and *GSTM1* on bronchial responsiveness in the European Community Respiratory Health Survey II, after adjustment for potential confounders considered *a priori* to be of relevance.

**Gas cooking-*GSTM1* interaction adjusted for:**	**Beta (95% CI)**
Unadjusted	-0.75 (-1.16 to -0.33)
Age (years) – sex interaction	-0.77 (-1.19 to -0.36)
Height (cm)	-0.77 (-1.18 to -0.36)
Smoking (pack-years)	-0.73 (-1.15 to -0.32)
Specific IgE titre (cat, house dust mite, Timothy-grass, *Cladosporium herbarum*) (< 0.35 vs ≥ 0.35 kU/L)	-0.70 (-1.10 to -0.29)
Log total IgE (kU/L)	-0.70 (-1.11 to -0.29)
Baseline FEV1	-0.83 (-1.23 to -0.43)
FEV1/FVC	-0.74 (-1.14 to -0.35)
GSTM1 – smoking interaction	-0.76 (-1.17 to -0.34)
GSTT1 and *GSTP1*-rs1695	-0.68 (-1.16 to -0.20)

Supplementary T4. Estimates for the interaction between gas cooking and genetic variants in *GSTM1*, *GSTT1*, and *GSTP1* on bronchial responsiveness, by sex, in the European Community Respiratory Health Survey II.

	**Males**			**Females**		
**Genetic variant**	**N**	**Beta (95% CI)**	***P***	**N**	**Beta (95% CI)**	***P***
***GSTM1* null**	1,086	-0.79 (-1.63 to 0.06)	0.067	1,122	-0.79 (-1.39 to 0.19)	0.010
***GSTT1* null**	1,086	0.42 (-0.56 to 1.39)	0.401	1,122	-0.55 (-1.28 to 0.18)	0.138
***GSTP1*-rs1695**	1,208	-0.30 (-0.87 to 0.27)	0.298	1,236	0.34 (-0.27 to 0.94)	0.271

Supplementary T5. Prevalence of gas cooking, as compared to cooking with electricity, and *GSTM1* null genotype in the European Community Respiratory Health Survey.

	**Gas cooking**		***GSTM1* null**	
**Country**	ECRHS I	ECRHS II	ECRHS I	ECRHS II
**Australia**	83.5%	84.9%	52.3%	50.6%
**Belgium**	53.9%	-	52.8%	-
**Estonia**	-	43.5%	-	43.5%
**France**	71.9%	64.4%	51.9%	51.5%
**Germany**	35.2%	12.4%	51.4%	53.8%
**Spain**	84.2%	54.8%	49.1%	49.0%
**Switzerland**	44.6%	22.1%	49.3%	57.5%
**UK**	61.8%	53.3%	54.0%	53.3%
**Combined**	66.5%	50.6%	51.2%	51.2%

Note: After exclusion of subjects with missing data on cooking fuel or not using gas or electric stove (ECRHS I: N = 83; ECRHS II: N = 71), who did not perform the methacholine challenge test (ECRHS I - Estonia: N = 215; ECRHS II - Belgium: N = 503) or had missing data on BHR slope (ECRHS I: N =742; ECRHS II: N = 675), and those with missing data on at least one of terms in the statistical model (ECRHS I: N = 325; ECRHS II: N = 191). Three hundred and fourteen subjects from ECRHS I and three hundred and thirty eight subjects from ECRHS II have missing data on *GSTM1* genotype.

Supplementary T6. Characteristics of subjects from the European Community Respiratory Health Survey not included versus those included in the final analysis.

	**Not included in the analysis (N = 5,901)**	**Included in the analysis (N = 2,208)**	***P****
**Age (mean, standard deviation)**	42.6 (7.1)	42.3 (7.3)	0.066
**Sex (%)**			
Males	46.3%	49.2%	0.020
Females	53.7%	50.8%	
**Smoking status (%)**[a]			
Never smoker	46.4%	44.3%	0.102
Ever smoker	53.6%	55.7%	
**Smoking pack-years (mean, standard deviation)**[a]	10.0 (16.2)	10.8 (17.6)	0.056
**Cooking fuel (%)**[b]			
Electricity	48.1%	49.5%	0.308
Gas	51.9%	50.5%	
**Bronchial responsiveness, log-slope (mean, standard deviation)**[c]	7.5 (2.2)	7.4 (2.3)	0.315

P was calculated using Pearson's chi-square test for count data and Student's t-test for continuous data.

[a]Three hundred and eighty two non-included subjects have missing information on smoking status and pack-years.

[b]Norway and Sweden were excluded from the analysis for exclusive cooking with electricity, and two hundred and eighty two non-included subjects have missing data on cooking fuel.

[c]Two thousand two hundred and eighty seven non-included subjects either did not perform methacholine test or have missing information on bronchial responsiveness.

Supplementary T7. Characteristics of genotyped subjects from the European Community Respiratory Health Survey not included versus those included in the final analysis.

	**Genotyped not included in the analysis (N = 2,857)**	**Genotyped included in the analysis (N = 2,208)**	***P****
**Age (mean, standard deviation)**	43.0 (7.2)	42.3 (7.3)	< 0.001
**Sex (%)**			
Males	46.8%	49.2%	0.087
Females	53.2%	50.8%	
**Smoking status (%)**[a]			
Never smoker	46.5%	44.3%	0.139
Ever smoker	53.5%	55.7%	
**Smoking pack-years (mean, standard deviation)**[a]	10.2 (17.2)	10.8 (17.6)	0.271
**Cooking fuel (%)**[b]			
Electricity	50.6%	49.5%	0.495
Gas	49.4%	50.5%	
GSTM1[c]			
Present	49.1%	48.8%	0.851
Null	50.9%	51.2%	
**Bronchial responsiveness, log-slope (mean, standard deviation)**[d]	7.6 (2.2)	7.4 (2.3)	0.058

***P** was calculated using Pearson's chi-square test for count data and Student's t-test for continuous data.

[a]Two hundred and thirteen non-included subjects have missing information on smoking status and pack-years.

[b]Norway and Sweden were excluded from the analysis for exclusive cooking with electricity, and seventy one non-included subjects have missing data on cooking fuel.

[c]Five hundred and ninety seven non-included subjects have missing data on *GSTM1* genotype.

[d]One thousand three hundred and eighty two non-included subjects either did not perform methacholine test or have missing information on bronchial responsiveness.
